# Ophthalmic manifestations of celiac disease in Tabuk City, Saudi Arabia

**DOI:** 10.3389/fmed.2026.1801404

**Published:** 2026-09-08

**Authors:** Seham S. Alhemaidi, Omnia S. Elseifi, Faris Hashem, Maisa Elhalabi, Naif M. Alali, Hani B. ALBalawi, Maher F. Al-Juhani, Sumayah A. Alzahrani, Albaraa A. Altowijri

**Affiliations:** 1 Division of Ophthalmology, Department of Surgery, Faculty of Medicine, University of Tabuk, Tabuk, Saudi Arabia; 2 Department of Family and Community Medicine, Faculty of Medicine, University of Tabuk, Tabuk, Saudi Arabia; 3 Pediatrics Department, King Salman Armed Forces Hospital in Northwestern Region, Tabuk, Saudi Arabia; 4 Ophthalmology Department, King Salman Armed Forces Hospital in Northwestern Region, Tabuk, Saudi Arabia; 5 Department of Surgery, Faculty of Medicine, University of Tabuk, Tabuk, Saudi Arabia

**Keywords:** autoimmune diseases, celiac disease, epidemiology, eye manifestations, retinal diseases

## Abstract

**Background:**

Celiac disease (CD) is an autoimmune enteropathy affecting approximately 0.7–1.4% of the global population. However, extraintestinal manifestations of CD, particularly ophthalmic conditions, remain underrecognized. This study aims to describe the spectrum and frequency of ophthalmic manifestations among a cohort of patients with CD in Tabuk, Saudi Arabia.

**Methods:**

This 5-year retrospective observational study was conducted at a tertiary care center and included 83 patients with CD, with no age restrictions. Patients were identified through the hospital’s electronic medical records using a diagnosis of CD established by a gastroenterologist based on standard clinical, serological, and/or histopathological criteria. Ophthalmic manifestations were retrieved from comprehensive ophthalmic examinations documented by ophthalmologists. For analysis, participants were stratified into pediatric (≤14 years) and adult (>14 years) groups based on their age at the time of data collection.

**Results:**

Ophthalmic manifestations were identified in 37.3% (95% CI: 27.0 to 48.7%) of the reviewed patient records. The most common ocular manifestations included refractive errors (12, 95% CI: 5.9 to 21.0%), followed by allergic conjunctivitis and dry eye disease (each 4.8, 95% CI: 1.3 to 11.9%). No significant associations were observed between age groups or diabetes mellitus and the prevalence of individual ophthalmic diagnoses. However, when ophthalmic involvement was analyzed as a composite outcome (defined by the presence of any ophthalmic disorder), a multivariable logistic regression analysis demonstrated that older age (adjusted OR: 3.25, 95% CI: 1.09 to 9.66, *p* = 0.034) and diabetes mellitus (adjusted OR: 3.32, 95% CI: 1.17 to 9.44, *p* = 0.024) were independently associated with increased odds of having ophthalmic involvement.

**Conclusion:**

In this cohort, ophthalmic findings were documented in over one-third of patients with CD. However, the prevalence of refractive errors was comparable to that reported in the general population, and due to the absence of a control group, a disease-specific association between CD and ophthalmic manifestations could not be established. Nevertheless, adults and patients with co-occurring diabetes demonstrated a higher likelihood of ocular involvement. These results highlight the need to raise awareness of ocular findings in patients with CD among healthcare professionals. Future controlled studies, particularly matched case–control or prospective cohort designs, are recommended to better assess these potential associations.

## Introduction

Celiac disease (CD) is an autoimmune enteropathy that remains a major health problem worldwide (1). A meta-analysis of patients with CD revealed an estimated global prevalence of 0.7–1.4%, with a rate of approximately 0.6% in Asia (2). Wide variations in the prevalence of CD have been reported, ranging from 0.14 to 3.2% across the Arab countries. A systematic review of regional studies revealed that the highest prevalence of CD among otherwise healthy individuals was reported in Saudi Arabia, at 3.2% (3).

Clinically, CD may present as an asymptomatic disease or manifest with gastrointestinal and/or extraintestinal symptoms. The most frequent extraintestinal manifestations (EIMs) associated with CD include anemia, osteoporosis, neurological disorders, dermatitis herpetiformis, and ophthalmic disorders (1, 4). Antigenic mimicry, circulating auto-antibodies, proinflammatory cytokines, and nutritional deficiencies contribute to the development of these EIMs in patients with CD (4). The onset of EIMs is observed in the majority of patients with CD, irrespective of age. More than 50% of patients with CD report EIMs in CD at the time of diagnosis, which occur without accompanying intestinal manifestations in 9% of cases (5).

However, ophthalmic manifestations reported in patients with CD are less common than other EIMs and are primarily related to either nutrient malabsorption (e.g., vitamin A, vitamin D, and calcium) or immune-mediated mechanisms (1). Recently, an accumulating body of evidence has linked CD with various ophthalmic disorders. Epidemiological studies have demonstrated that patients with CD have a 1.32 times higher risk of uveitis (HR: 1.32; 95% CI 1.10 to 1.58) (6) and an approximately fivefold higher risk of cataracts than the general population (7, 8). Disorders of the posterior segment have also been reported, with observed changes in retinal and choroidal morphology (8–11). Moreover, secondary effects induced by vitamin deficiencies have been reported among CD patients (8), such as keratomalacia and corneal ulceration, due to vitamin A malabsorption (12). These extraintestinal ophthalmic findings emphasize the need for interdisciplinary awareness, routine ophthalmic evaluation of symptomatic patients with CD, and early referral to prevent vision loss.

With the rising prevalence of CD in Saudi Arabia, especially among asymptomatic children, it is important that EIMs associated with CD be screened and treated at an early stage of the disease. However, ophthalmic manifestations in patients with CD and their prevalence among adults and children have not been well-studied in the Saudi population. Thus, ophthalmic manifestations in patients with CD may be underrecognized by healthcare practitioners (13). Therefore, to address this unmet need, we conducted this retrospective chart review study to describe the spectrum and frequency of ophthalmic manifestations observed among pediatric and adult patients with CD in Tabuk, Saudi Arabia. Our findings provide valuable insights into the spectrum range of ophthalmic findings encountered in routine clinical practice, enhance clinical awareness of potential ocular involvement, and highlight priority areas for further prospective research.

## Methods

### Ethical considerations

The procedures followed were conducted in accordance with the ethical standards of the Declaration of Helsinki, as revised in 2000, and were approved by the Research Ethics Committees of the University of Tabuk and King Salman Armed Forces Hospital in Northwestern Region.

#### Study design, settings, and participants

A 5-year retrospective observational study was conducted, including all children and adult patients diagnosed with CD at King Salman Armed Forces Hospital, Tabuk, between 2015 and 2020. Patients were identified through the hospital’s electronic medical records database using documented diagnoses of CD established by the treating gastroenterologist according to standard clinical, serological, and/or histopathological criteria. However, patients with CD who had known congenital eye conditions were excluded, whereas acquired ophthalmic conditions documented in the medical records before or at the time of evaluation were included in the analysis. Patients were categorized into pediatric (≤14 years) and adult age groups according to their age at the time of data collection.

Ophthalmic manifestations were ascertained through a retrospective review of electronic medical records. Diagnoses were based on documented ophthalmic findings recorded during routine clinical care. No standardized ophthalmological examination or systematic referral to an ophthalmologist was performed as part of the study.

#### Data collection

A total of 83 eligible patients were identified. The de-identified medical records of all 83 patients with CD were reviewed by the authors on 28 September 2021, and the variables of interest were recorded and entered into an Excel spreadsheet. The variables of interest included age group (pediatric and adult groups), sex, age at CD diagnosis, presence of symptoms suggestive of ophthalmic complications, and presence of signs suggestive of ocular involvement.

All selected charts included a comprehensive ophthalmological examination performed and documented by ophthalmology specialists as part of routine clinical care. The charts included medical history, visual acuity, refraction (for refractive errors), intraocular pressure (IOP) measurements using a Tono-Pen tonometer (for glaucoma), slit-lamp examination findings (for conjunctivitis, cataracts, and uveitis), and fundus examination findings (for papilledema). Diabetes status was recorded as present or absent based on a documented physician diagnosis in the medical record.

The primary outcome was the presence of ophthalmic manifestations, defined as the occurrence of one or more abnormal ophthalmological findings or diagnoses documented during the study period.

#### Statistical analysis

Categorical data were expressed as frequencies and percentages. The prevalence of ophthalmic manifestations was calculated with corresponding exact 95% confidence intervals (CIs). The potential associations between the studied variables and age groups and diabetes status were assessed using Pearson’s chi-square test or Fisher’s exact test, as appropriate. Differences in proportions were reported alongside their 95% CIs and *p-*values. To identify independent risk factors associated with the presence of ophthalmic manifestations, logistic regression analysis was performed. Variables included in the multivariable model were selected *a priori* based on their clinical relevance and potential confounding effects reported in the previous literature. Specifically, age group and diabetes status were included as primary explanatory variables, and sex was included as an adjustment variable. Adjusted odds ratios (aORs) and 95% CIs were calculated. A two-sided *p-*value of < 0.05 was considered statistically significant for all tests. The *p-*values were additionally adjusted using the Benjamini–Hochberg false discovery rate procedure. All analyses were conducted using R statistical software (R Foundation for Statistical Computing, Vienna, Austria; version 4.5.3) (14).

## Results

### Patient characteristics

A total of 83 patients with CD were included during the study period (2015–2020). Of these, 66.3% of patients were female and 33.7% were male. The mean age was 23.3 ± 14 years, with a range of 3 to 58 years. Overall, 61.4% of the participants were adults (mean age 31.3 ± 11.9 years, range: 15 to 58 years), whereas only 38.6% were pediatric patients (mean age: 10.4 ± 2.9 years, range: 3 to 14 years; Table 1).

**Table 1:** Distribution of systemic diagnoses associated with celiac disease according to patients’ age groups.

Characteristic^a^	Total *N* = 83	Pediatrics *N* = 32	Adults *N* = 51	Difference (95% CI)	*p*-value^b^	FDR-adjusted *p*-value
Age (years), Mean ± SD (range)	23.3 ± 14.0 (3.0–58.0)	10.4 ± 2.9 (3.0–14.0)	31.3 ± 11.9 (15.0–58.0)	–	–	–
Celiac disease only, *n* (%)	29 (34.9%)	6 (18.8%)	23 (45.1%)	−26.3% (−48.1 to −4.6%)^c^	0.014*^c^	0.048*^e^
Celiac with other associated diseases, *n* (%)	54 (65.1%)	26 (81.3%)	28 (54.9%)	26.3% (4.6 to 48.1%)^c^		
Diabetes mellitus, *n* (%)	43 (51.8%)	24 (75.0%)	19 (37.3%)	37.7% (15.2 to 60.3%)^c^	<0.001*^c^	0.008*^e^
Hypothyroidism, *n* (%)	11 (13.3%)	2 (6.3%)	9 (17.6%)	−11.4% (−27.3 to 4.6%)^c^	0.190^d^	0.380^e^
Iron deficiency anemia, *n* (%)	6 (7.2%)	3 (9.4%)	3 (5.9%)	3.5% (−11.0 to 18.0%)^c^	0.672^d^	0.840^e^
Autoimmune thyroiditis, *n* (%)	3 (3.6%)	0 (0.0%)	3 (5.9%)	−5.9% (−14.9 to 3.1%)^c^	0.281^d^	0.468^e^
Bronchial asthma, *n* (%)	2 (2.4%)	1 (3.1%)	1 (2.0%)	1.2% (−7.1 to 9.5%)^c^	>0.999^d^	>0.999^e^
Down syndrome, *n* (%)	2 (2.4%)	2 (6.3%)	0 (0.0%)	6.3% (−4.7 to 17.2%)^c^	0.146^d^	0.364^e^
Cardiac diseases, *n* (%)	1 (1.2%)	1 (3.1%)	0 (0.0%)	3.1% (−5.4 to 11.7%)^c^	0.386^d^	0.551^e^
Sickle cell disease, *n* (%)	1 (1.2%)	0 (0.0%)	1 (2.0%)	−2.0% (−7.7 to 3.8%)^c^	>0.999^d^	>0.999^e^

CI = confidence interval; FDR = false discovery rate.

a CI: confidence interval; *n*: number; SD: standard deviation.

b * Significant at a *p*-value of <0.05.

c Pearson’s chi-squared test.

d Fisher’s exact test.

e Benjamini–Hochberg correction for multiple testing.

Nearly 34.9% of patients had isolated CD, whereas 65.1% had at least one associated comorbidity. The most common associated comorbidity was diabetes mellitus (51.8%), followed by hypothyroidism (13.3%), and the least common were sickle cell anemia and cardiac diseases (1.2%) for each.

Comparison by age group showed that isolated CD was more common among adults, whereas CD associated with other comorbidities was significantly more frequent among pediatric patients (difference: 26.3, 95% CI: 4.6–48.1%; *p* = 0.014). Similarly, diabetes mellitus was significantly more prevalent among pediatric patients than among adults (difference: 37.7, 95% CI: 15.2–60.3%; *p* < 0.001; Table 1). The higher prevalence of diabetes observed in pediatric patients is likely attributable to type 1 DM, which is more common in this age group.

### Prevalence of ophthalmic manifestations

Ophthalmic manifestations were identified in 31 of the 83 patients, corresponding to a prevalence of 37.3% (95% CI: 27.0–48.7%). The most frequently reported ophthalmic finding was refractive error, occurring in 12% (95% CI: 5.9–21.0%). Allergic conjunctivitis and dry eye disease were the second most common manifestations, each affecting 4.8% of patients (95% CI: 1.3–11.9%). Unspecified retinal abnormalities (not attributable to DM) were recorded in 3.6% of patients (95% CI: 0.8–10.2%), whereas proliferative diabetic retinopathy, cataract, and uveitis were each observed in 2.4% of patients (95% CI: 0.3–8.4%). Non-proliferative diabetic retinopathy, glaucoma, esotropia, and papilledema were the least common findings, each occurring in 1.2% of patients (95% CI: 0.0–6.5%; Table 2).

**Table 2:** The associated ophthalmic manifestations among participants.

Characteristic^a^	Total *N* = 83	95% CI
Ophthalmic manifestations, *n* (%)		
Absent	52 (62.7%)	51.3–73.0%
Refractive errors	10 (12.0%)	5.9–21.0%
Allergic conjunctivitis	4 (4.8%)	1.3–11.9%
Dry eye disease	4 (4.8%)	1.3–11.9%
Non-diabetic retinal abnormalities^b^	3 (3.6%)	0.8–10.2%
Proliferative diabetic retinopathy	2 (2.4%)	0.3–8.4%
Uveitis	2 (2.4%)	0.3–8.4%
Cataract	2 (2.4%)	0.3–8.4%
Non-proliferative diabetic retinopathy	1 (1.2%)	0.0–6.5%
Glaucoma	1 (1.2%)	0.0–6.5%
Esotropia	1 (1.2%)	0.0–6.5%
Papilledema	1 (1.2%)	0.0–6.5%

CI = confidence interval.

a CI: confidence interval; *n*: number.

b “Non-diabetic retinal abnormalities” refers to retinal abnormalities judged by the treating ophthalmologist to be unrelated to diabetic retinal disease based on the documented clinical diagnosis (e.g., retinal vasculitis and anemic retinopathy), regardless of whether the patient had diabetes mellitus.

### Subgroup analyses

The prevalence of ophthalmic manifestations in patients with CD was higher among adults than among pediatric patients (difference: 15.0, 95% CI: −8.2 to 38.2%). However, this association did not reach statistical significance (*p* = 0.169). The wide confidence interval suggests limited precision in the estimate, likely due to the relatively small sample size. Similarly, no significant association was observed between age group and any individual ophthalmic manifestation (all *p* > 0.05; Table 3).

**Table 3:** Distribution of ophthalmic manifestations according to age groups.

Characteristic^a^	Total *N* = 83	Pediatrics *N* = 32	Adults *N* = 51	Difference (95% CI)	*p*-value^b^	FDR-adjusted *p*-value
Absent, *n* (%)	52 (62.7%)	23 (71.9%)	29 (56.9%)	15.0% (−8.2 to 38.2%)^c^	0.169^d^	>0.999^f^
Allergic conjunctivitis, *n* (%)	4 (4.8%)	0 (0.0%)	4 (7.8%)	−7.8% (−17.8 to 2.1%)^c^	0.734^e^	>0.999^f^
Dry eye disease, *n* (%)	4 (4.8%)	1 (3.1%)	3 (5.9%)	−2.8% (−14.1 to 8.6%)^c^	0.156^e^	>0.999^f^
Non-diabetic retinal abnormalities, *n* (%) ^g^	3 (3.6%)	2 (6.3%)	1 (2.0%)	4.3% (−7.5 to 16.0%)^c^	>0.999^e^	>0.999^f^
Proliferative diabetic retinopathy, *n* (%)	2 (2.4%)	1 (3.1%)	1 (2.0%)	1.2% (−7.1 to 9.5%) ^c^	0.556^e^	>0.999^f^
Uveitis, *n* (%)	2 (2.4%)	0 (0.0%)	2 (3.9%)	−3.9% (−11.8 to 3.9%)^c^	>0.999^e^	>0.999^f^
Cataract, *n* (%)	2 (2.4%)	1 (3.1%)	1 (2.0%)	1.2% (−7.1 to 9.5%)^c^	0.520^e^	>0.999^f^
Refractive errors, *n* (%)	10 (12.0%)	3 (9.4%)	7 (13.7%)	−4.4% (−20.7 to 12.0%)^c^	>0.999^e^	>0.999^f^
Non-proliferative diabetic retinopathy, *n* (%)	1 (1.2%)	0 (0.0%)	1 (2.0%)	−2.0% (−7.7 to 3.8%)^c^	>0.999^e^	>0.999^f^
Glaucoma, *n* (%)	1 (1.2%)	0 (0.0%)	1 (2.0%)	−2.0% (−7.7 to 3.8%)^c^	>0.999^e^	>0.999^f^
Esotropia, *n* (%)	1 (1.2%)	1 (3.1%)	0 (0.0%)	3.1% (−5.4 to 11.7%)^c^	0.386^e^	>0.999^f^
Papilledema, *n* (%)	1 (1.2%)	0 (0.0%)	1 (2.0%)	−2.0% (−7.7 to 3.8%)^c^	>0.999^e^	>0.999^f^

CI = confidence interval; FDR = false discovery rate.

a CI: confidence interval; *n*: number.

b *Significant at *p* < 0.05.

c Absolute difference in proportions between pediatric and adult groups.

d Pearson’s chi-squared test.

e Fisher’s exact test.

f Benjamini–Hochberg correction for multiple testing.

g “Non-diabetic retinal abnormalities” refers to retinal abnormalities judged by the treating ophthalmologist to be unrelated to diabetic retinal disease based on the documented clinical diagnosis (e.g., retinal vasculitis and anemic retinopathy), regardless of whether the patient had diabetes mellitus.

Some ophthalmic conditions were exclusively recorded among patients with diabetes mellitus, including cataracts, esotropia, and diabetic retinopathy. In addition, refractive errors were more common among patients with diabetes mellitus than among those without diabetes mellitus. However, no significant association was observed between the presence of diabetes mellitus and any individual ophthalmic disorder (all *p* > 0.05; Table 4). Non-diabetic retinal abnormalities were identified in three patients (3.6%), all of whom had diabetes mellitus. These findings included retinal vasculitis and anemic retinopathy and were classified as retinal conditions unrelated to diabetic retinopathy based on documented ophthalmological diagnoses.

**Table 4:** Distribution of ophthalmic manifestations according to the presence of diabetes mellitus.

Characteristic^a^	Total *N* = 83	Non-diabetic *N* = 40	Diabetic *N* = 43	Difference (95% CI)	*p*-value^b^	FDR-adjusted *p*-value
Absent, *n* (%)	52 (62.7%)	29 (72.5%)	23 (53.5%)	19.0% (−3.7 to 41.8%)^c^	0.074^d^	0.391^f^
Allergic conjunctivitis, *n* (%)	4 (4.8%)	3 (7.5%)	1 (2.3%)	5.2% (−6.6 to 16.9%)^c^	0.348^e^	0.643^f^
Dry eye disease, *n* (%)	4 (4.8%)	3 (7.5%)	1 (2.3%)	5.2% (−6.6 to 16.9%)^c^	0.348^e^	0.643^f^
Non-diabetic retinal abnormalities, *n* (%)^g^	3 (3.6%)	0 (0.0%)	3 (7.0%)	−7.0% (−17.0 to 3.1%)^c^	0.242^e^	0.629^f^
Proliferative diabetic retinopathy, *n* (%)	2 (2.4%)	0 (0.0%)	2 (4.7%)	−4.7% (−13.4 to 4.1%)^c^	0.495^e^	0.643^f^
Uveitis, *n* (%)	2 (2.4%)	2 (5.0%)	0 (0.0%)	5.0% (−4.2 to 14.2%)^c^	0.229^e^	0.629^f^
Cataract, *n* (%)	2 (2.4%)	0 (0.0%)	2 (4.7%)	−4.7% (−13.4 to 4.1%)^c^	0.495^e^	0.643^f^
Refractive errors, *n* (%)	10 (12.0%)	2 (5.0%)	8 (18.6%)	−13.6% (−29.5 to 2.3%)^c^	0.090^e^	0.391^f^
Non-proliferative diabetic retinopathy, *n* (%)	1 (1.2%)	0 (0.0%)	1 (2.3%)	−2.3% (−9.2 to 4.5%)^c^	>0.999^e^	>0.999^f^
Glaucoma, *n* (%)	1 (1.2%)	0 (0.0%)	1 (2.3%)	−2.3% (−9.2 to 4.5%)^c^	>0.999^e^	>0.999^f^
Esotropia, *n* (%)	1 (1.2%)	0 (0.0%)	1 (2.3%)	−2.3% (−9.2 to 4.5%)^c^	>0.999^e^	>0.999^f^
Papilledema, *n* (%)	1 (1.2%)	1 (2.5%)	0 (0.0%)	2.5% (−4.8 to 9.8%)^c^	0.482^e^	0.643^f^

CI = confidence interval; FDR = false discovery rate.

a CI: confidence interval; *n*: number.

b *Significant at *p* < 0.05.

c Absolute difference in proportions between diabetic and non-diabetic groups.

d Pearson’s chi-squared test.

e Fisher’s exact test.

f Benjamini–Hochberg correction for multiple testing.

g ^“^Non-diabetic retinal abnormalities” refers to retinal abnormalities judged by the treating ophthalmologist to be unrelated to diabetic retinal disease based on the documented clinical diagnosis (e.g., retinal vasculitis and anemic retinopathy), regardless of whether the patient had diabetes mellitus.

After adjusting for multiple comparisons using the Benjamini–Hochberg procedure, the statistical significance of the reported associations remained unchanged.

### Multivariable analysis

A logistic regression analysis was conducted to assess participant characteristics significantly associated with ophthalmic involvement as a composite outcome (defined as the presence of any ophthalmic disorder). Univariable analyses showed no significant associations. In contrast, the multivariable model demonstrated that adult patients were approximately three times more likely to have ophthalmic involvement than pediatric patients (adjusted OR: 3.25, 95% CI: 1.09–9.66, *p* = 0.034), after adjusting for sex and diabetic status. Similarly, patients with diabetes had a threefold increased likelihood of experiencing ophthalmic manifestations compared with those without diabetes, after adjusting for age group and sex (adjusted OR: 3.32, 95% CI: 1.17 to 9.44, *p* = 0.024; Table 5).

**Table 5:** Logistic regression analysis for the presence of ophthalmic manifestations.

Characteristic	Unadjusted OR (95% CI)	*p*-value^a^	Adjusted OR (95% CI)	*p*-value^a^
Age groups				
Pediatrics (*n* = 32)	–		–	
Adults (*n* = 51)	1.89 (0.73–4.85)	0.188	3.25 (1.09–9.66)	0.034*
Sex				
Male (*n* = 28)	–		–	
Female (*n* = 55)	0.57 (0.22–1.44)	0.232	0.54 (0.20–1.45)	0.218
Systemic diagnoses				
Celiac disease only (*n* = 29)	–			
Celiac with other associated diseases (*n* = 54)	1.89 (0.71–4.98)	0.200		
Diabetes mellitus (*n* = 43)	2.24 (0.90–5.58)	0.084	3.32 (1.17–9.44)	0.024*
Hypothyroidism (*n* = 11)	0.64 (0.16–2.56)^b^	0.532		
Iron deficiency anemia (*n* = 6)	3.31 (0.58–18.72)^b^	0.177		

CI = confidence interval, OR = odds ratio.

Multivariable model: observations = 83; AICc = 108; Nagelkerke pseudo-*R*^2^ = 0.154.

a *p < 0.05.

b ORs for hypothyroidism and iron deficiency anemia are presented from the univariable analysis only and were not included in the multivariable model due to lack of statistical significance and considerations of model parsimony given the sample size.

## Discussion

In the past few decades, the prevalence of CD has increased worldwide. With the increasing prevalence of CD in the Gulf countries, particularly Saudi Arabia, additional studies are needed to better define the true burden and determinants of CD and its EIMs.

In the current study, the sex distribution of CD was higher among female patients (66.3%) than among male patients (33.7%). These findings are consistent with those of a global epidemiological study, which reported that CD is 1.5 times more common in female patients than in male patients (2). However, the same study estimated that CD is twice as common in children as in adults, unlike the findings of the present study, in which 61.4% of patients with CD were adults (2). These differences in age groups should be interpreted cautiously, as they may reflect true differences in disease recognition, referral patterns, and diagnostic rates rather than true differences in disease burden or ophthalmic manifestations. Furthermore, many patients with CD (particularly children) in Saudi Arabia and the Gulf countries remain undiagnosed (3, 13), with 1% of asymptomatic Saudi children reported to have CD, suggesting that the prevalence of CD in Saudi children may be underestimated (15).

Being an autoimmune disease, CD is often associated with other systemic conditions, particularly diabetes mellitus. In our study, more than 50% of the patients with CD had diabetes mellitus, consistent with previous reports demonstrating the frequent co-occurrence of diabetes mellitus and CD (16–19). This close association between diabetes and CD may result from genetic predisposition, viral infections, diet, and alterations in the gut microbiome, all of which have been implicated in the pathogenesis of both diseases (20). Other commonly associated conditions include hypothyroidism, sickle cell anemia, and cardiac diseases. Due to its diverse manifestations, patients with CD often present to healthcare professionals other than gastroenterologists or pediatricians. Therefore, patients with these associated conditions should be referred for CD evaluation.

In this study, the most common ocular manifestations were refractive errors (12, 95% CI: 5.9 to 21.0%), allergic conjunctivitis (4, 95% CI: 1.3 to 11.9%), and dry eye disease (4, 95% CI: 1.3 to 11.9%). These findings were in line with those of a large cross-sectional descriptive study, in which dry eye (32%) and cataracts (12%) were the most common ophthalmic diseases in patients with CD (1). However, the present study did not include a control group, making it impossible to determine whether the observed prevalence of ophthalmic findings (37.3%) was higher than that observed in the general population. Accordingly, these findings should be interpreted as the frequency of ophthalmic conditions within our CD cohort rather than as evidence of a disease-specific association, particularly because these disorders are highly prevalent in the general population. A comparison of the prevalence of refractive errors among our patients with that reported in the general population showed comparable rates. Estimates from community surveys report a prevalence of 10–18% for myopia, 2–14% for hyperopia, and 10–45% for astigmatism in the general population (21, 22). Similarly, allergic conjunctivitis is a widespread condition, with prevalence rates ranging from 5 to 45%, depending on geographic, environmental, and demographic factors (23). Therefore, while our results highlight the presence of common ophthalmic conditions among patients with CD, they do not establish a disease-specific association. Future controlled or population-based studies are warranted to clarify whether CD is associated with an increased risk of these manifestations.

Clinicians should be aware that patients with CD undergoing corneal refractive surgery may require careful preoperative ocular surface evaluation because previous studies have reported possible corneal alterations, although the evidence remains inconclusive (9, 24–26).

In the present study, the multivariable regression analysis of the presence of ophthalmic conditions revealed a significantly higher likelihood of ophthalmic involvement among patients with diabetes than among those without diabetes mellitus. The emergence of statistical significance in the multivariable model, despite non-significant univariable associations, may reflect confounding and the effect of covariate adjustment, which can reveal associations not evident in unadjusted analyses. The multivariable model demonstrated a Nagelkerke pseudo-*R*^2^ of 0.154, indicating modest explanatory power, which is expected in clinical observational studies and reflects the multifactorial etiology of ophthalmic manifestations. These results suggest that some of the observed ophthalmic disorders may be related to diabetes mellitus rather than CD. Confirmation of this hypothesis requires further studies with larger sample sizes to account for additional confounders and to conduct separate regression analyses for each ophthalmic diagnosis. All cases categorized as “non-diabetic retinal abnormalities” occurred in patients with diabetes mellitus. This apparent overlap should not be interpreted as diabetic retinopathy, as these findings were clinically judged by the treating ophthalmologists to be unrelated to diabetic retinal disease. Instead, they represented alternative retinal pathologies, including retinal vasculitis and anemic retinopathy. This distinction highlights that not all retinal abnormalities in patients with diabetes are attributable to diabetic retinopathy and underscores the importance of etiological classification based on clinical ophthalmological assessment.

The literature indicates that vitamin deficiencies, oxidative stress, and other autoimmune diseases, such as diabetes, increase the risk of cataract development in patients with CD (12). One study reported that the absolute risk of cataracts was 397/100,000 person-years among patients with CD, with an excess risk of 86/100,000 person-years (7). Although our study reported cataracts in only 2.4% of patients, this finding is noteworthy considering the small sample size and the wide age range of participants included in the study.

The current study also reported a few cases of uveitis. A moderately increased risk of uveitis among patients with biopsy-verified CD has also been reported (HR: 1.32; 95% CI: 1.10–1.58) (6). Adherence to a gluten-free diet has been associated with significant improvement in cases of cataracts and uveitis (6, 7). Many of these ophthalmic conditions may appear prior to the confirmatory diagnosis of CD. Therefore, it is important to evaluate patients for CD when they present with any of these conditions.

Diagnosis of ocular manifestations in pediatric patients with CD often requires time. Previous studies have reported that patients with CD may exhibit increased choroidal thickness, retinal nerve fiber layer thinning, anterior chamber shallowing, and qualitative and quantitative tear film abnormalities (9, 11, 27). These observations highlight the importance of maintaining clinical awareness of potential ocular manifestations. However, whether routine ophthalmological screening of asymptomatic pediatric patients with CD is beneficial remains to be established through prospective controlled studies.

To the best of our knowledge, no previous studies from Saudi Arabia have comprehensively assessed ophthalmic manifestations in patients with CD across both adult and pediatric populations. By documenting the range of ocular conditions encountered in routine clinical practice, our findings contribute to a more comprehensive characterization of potential ophthalmic involvement in this population.

However, a few limitations should be considered when interpreting the results. As a retrospective study, ocular findings were based on the clinical records of patients who underwent ophthalmological assessment, often prompted by symptoms or clinical indications. Accordingly, the reported prevalence reflects documented ophthalmic findings in this hospital-based cohort rather than the true prevalence among all patients with CD and may be subject to selection bias, potentially leading to either overestimation or underestimation. In addition, the single-center design limits the generalizability of the findings to other populations. The absence of a control group precludes the assessment of causal or disease-specific associations between CD and ophthalmic manifestations. Although the multivariable analysis identified age and diabetes as significant factors associated with ophthalmic findings, the outcome comprised heterogeneous ocular conditions, and the small number of events for individual diagnoses limits the interpretability of the composite outcome as a single clinically meaningful entity. Therefore, the observed associations may reflect background age-related ocular disease or diabetes-related pathology rather than CD-specific effects, and the relative contributions of CD, population baseline prevalence, and comorbidities cannot be distinguished. Finally, the relatively small sample size, particularly in the pediatric subgroup, limited the precision of the estimates and subgroup analyses, as reflected by wide confidence intervals. These findings therefore require confirmation in larger, controlled studies.

## Conclusion

In this cohort, ophthalmic manifestations were identified in over one-third of patients, with refractive errors and ocular surface conditions being the most commonly observed findings. Notably, the prevalence of refractive errors in our cohort was comparable to that reported in the general population; therefore, our findings do not support a disease-specific association between CD and refractive errors. These findings provide insight into the spectrum and frequency of ophthalmic abnormalities encountered among patients with CD in our region and highlight that ocular conditions are commonly documented in this patient population. However, because of the retrospective design, the relatively small sample size, and the absence of a control group, the study cannot determine whether these ophthalmic manifestations occur more frequently than expected in the general population or whether they are specifically associated with CD. Therefore, the findings should be interpreted with caution. Clinicians caring for patients with CD should remain aware of potential ophthalmic comorbidities, particularly in adults and individuals with diabetes mellitus, and an ophthalmological evaluation may be considered when clinically indicated. Future multicenter, prospective case–control or cohort studies across Saudi Arabia with adequately powered sample sizes are warranted to clarify the relationship between CD and ophthalmic manifestations. Such studies should include an appropriate control group and evaluate potential confounders and disease-related factors, including gluten-free diet adherence, disease duration and severity, serological markers, nutritional status, and other comorbidities, such as diabetes mellitus.

## Data Availability

The original contributions presented in the study are included in the article/supplementary material, further inquiries can be directed to the corresponding author.
